# Meta-core outcome set for randomized trials and clinical practice for curative surgical oncology: patient and healthcare consensus statement

**DOI:** 10.1093/bjsopen/zrag019

**Published:** 2026-03-24

**Authors:** Joel Tay, Jane Blazeby, Declan Devane, Yoon Loke, Aoife Lowery, Adam O’Neill, Catherine Robinson, Ceri Steele, Bilal Alkhaffaf, Jamie Kirkham

**Affiliations:** Centre for Biostatistics, The University of Manchester, Manchester, UK; NIHR Bristol Biomedical Research Centre, University Hospitals Bristol and Weston NHS Foundation Trust and University of Bristol, Bristol, UK; Health Research Board-Trials Methodology Research Network (HRB-TMRN), Galway, Ireland; School of Nursing and Midwifery, University of Galway, Galway, Ireland; Norwich Medical School, University of East Anglia, Norwich, UK; Discipline of Surgery, University of Galway, Galway, Ireland; Social Care and Society, School of Health Sciences, The University of Manchester, Manchester, UK; Social Care and Society, School of Health Sciences, The University of Manchester, Manchester, UK; Independent Cancer Patients’ Voice, London, UK; Faculty of Biology, Medicine and Health, The University of Manchester, Manchester Academic Health Science Centre, Manchester, UK; Department of Oesophago-Gastric and Bariatric Surgery, Salford Royal Hospital, Northern Care Alliance NHS Foundation Trust, Salford, UK; Division of Cancer Sciences, School of Medical Sciences, Faculty of Biology, Medicine and Health, The University of Manchester, Manchester, UK; Centre for Biostatistics, The University of Manchester, Manchester, UK

**Keywords:** COMET, cancer surgery, Delphi

## Abstract

**Background:**

The development and use of core outcome sets (COSs) have the potential to inform best practice in healthcare treatment options by facilitating data synthesis across studies. In surgical oncology, eight cancer-specific COSs exist, and these overlap in content. The development of a meta-COS that includes the core outcomes from existing cancer data sets would be applicable, as a minimum, across all cancer types in surgical oncology for use in effectiveness trials and clinical practice.

**Methods:**

The process of developing a meta-COS included identification of the existing COS and their discussion with 3 different stakeholder focus groups (surgeons/oncologists, other healthcare professionals, and patients and carers). Outcomes were identified through a review of the literature and registered clinical trials. Stakeholders representing healthcare professionals (surgeons/oncologists and others) were recruited from national societies, regional cancer networks, and local trial centres, whereas patients and carers were recruited using independent representative organizations. The aim was to include at least two individuals from each group for the ten common cancers requiring surgery. These ten cancer types were regarded as the commonest based on global incidence in World Cancer Research Fund data. An agreed-upon list of outcomes was scored in a two-round online Delphi survey (March–May 2025). The results of this survey were discussed and ratified at an online consensus meeting (June 2025) by Delphi participants who completed both rounds, members of the stakeholder groups, and patients. The 80% threshold defined consensus.

**Results:**

Forty-five participants completed both survey rounds, and 15 participated in the consensus meeting. Of 317 outcomes initially identified, 35 were discussed with stakeholders, 32 were scored in Delphi rounds, and consensus was achieved on eight core outcomes to include in the surgical oncology meta-COS: overall survival, disease-free survival, disease-specific survival, death related to surgery, delay to further treatment, completeness of tumour removal, overall quality of life, and serious adverse events.

**Conclusion:**

This meta-COS for surgical oncology offers a standard set of outcomes to be used in surgical oncology studies and clinical practice, regardless of cancer type.

## Teaser

This article describes the consensus-driven development process of a minimum set of outcomes that should be reported in cancer surgery trials across specialities. This does not detract from previous work within those specialities, but supplements them and, more importantly, assists in cancer trials where the cancer types are still without a core outcome set for reference.

## Introduction

Cancer remains the leading cause of death worldwide^1^, and although the use of multimodal therapies has improved prognosis if the disease is detected early, surgery remains the single independent treatment modality that offers a potential cure^2^ for most solid organ malignancies. Identifying the optimum surgical approach involves balancing the benefits of a radical oncological resection against the risk and impact of associated complications and physiological consequences^3^. To compare outcomes from surgical trials in a clinically meaningful manner, it is essential to address heterogeneity in outcomes, ensuring that the most important outcomes are consistently assessed.

Core outcome sets (COSs) have been defined as ‘an agreed standardized collection of outcomes which should be measured and reported, as a minimum, in all trials for a specific clinical area’^4^. The implementation of COSs has increasingly been recognized^5^ as a concept that improves data synthesis from multiple studies to optimize clinical decision-making. Early attempts at standardizing outcomes for all types of cancer and treatment modalities exist from the early 1980s but lack patient input^6^. Over the past decade, eight COSs for specific cancer types (prostate^7^, colorectal^8^, gastric^3^, oesophageal^9^, oropharyngeal (ear, nose, and throat)^10^, bladder^11^, lung^12^, and pancreatic^13^), with a focus on curative surgical intervention, have been developed. Many of these have shown reasonable adherence to the minimum standards^14^ for COS development, which includes the involvement of patients in determining which outcomes are important^15^. There remains a significant evidence gap in the availability of surgical oncology COSs for some of the most common cancers, such as breast, thyroid, liver, and cervical cancers. Although each of these existing COS studies varied in scope (different cancer types), a recent appraisal of these existing surgical oncology COSs has identified overlapping key domains pertaining to outcomes related to survival, adverse outcomes, and the impact on health-related quality of life^16^, paving the way for a COS for all cancer types with surgical intervention.

The overall aim of this study was to develop a surgical oncology meta-COS for any cancer type involving a surgical procedure, either alone or combined with other therapies, with curative intent. The study aims to define endpoints to be reported as a minimum in future effectiveness trials and clinical practice. A meta-COS with relevance to clinical practice would mean these outcomes would be practically measurable, and include patient-reported outcomes.

## Methods

**Fig. 1:**
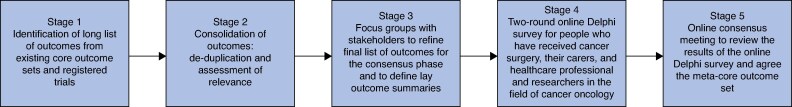
Process flow diagram of core outcome set development

The surgical oncology meta-COS project comprised five stages: identification of outcomes for consideration from the existing literature; a consolidation stage for determining which outcomes may be relevant for all cancer types; (3) focus groups with key stakeholders to finalize the list of outcomes and to provide lay outcome summaries for use in the consensus stages; a two-round online modified Delphi consensus process to rate the importance of the selected outcomes; and an online consensus building process to review and agree upon the final meta-COS. A process flow diagram illustrating these stages is provided in *Fig. 1*.

A study protocol was developed *a priori* using the COS-STAP recommendations^17^ and was approved by The University of Manchester Research Ethics Committee (Reference numbers 2024-21348-37826 and 2024-18269-33406) and made available^18^. The project was registered on the COMET database (https://www.comet-initiative.org/Studies/Details/3252) and is reported in line with the COS-STAR reporting guideline^19^, as shown in the *supplementary appendix*.

### Study group and participants

Four authors (JT, CR, BA, JK) oversaw the surgical oncology meta-COS project, identified and invited individuals with relevant expertise to form the core author group, and were responsible for the study methods and day-to-day project management.

The core author group had expertise in methodology, surgery (both cancer and general), and public and patient engagement. Two representatives joined the core group, one from the Independent Cancer Patients’ Voices (ICPV) and one from The University of Manchester, to advise on study methods, help provide patient-friendly resources (including lay outcome summaries), and support patient recruitment.

Stakeholders representing healthcare professionals (consultant surgeons or oncologists, cancer specialist nurses, and allied health professionals) were recruited from national societies, regional cancer networks and local trial centres, which included the British Association of Surgical Oncology, the North West Surgical Trials Unit, the National Breast Cancer Research Institute Ireland, the National Surgical Research Support Centre, Cancer Research UK, and the Greater Manchester Cancer Alliance. It was anticipated that many recruited participants from these networks would have some research experience, fulfilling the need to include researchers as part of the minimum standards for COS development^14^. Patients and carers were recruited using independent representative organizations (Greater Manchester Care Alliance, Patient and Public Involvement (PPI) Ignite Ireland, Irish Cancer Society, The Patients Association, ICPV, The Butterfly Thyroid Cancer Trust, National Breast Cancer Research Institute Ireland), as well as local networks through healthcare professionals.

The aim was to recruit at least two individuals from each stakeholder group (surgeons/oncologists, other healthcare professionals, and patients and carers) for the ten most common cancers by incidence^20^, providing an approximate sample size of 60 participants.

### Stage 1: identification of outcomes

As a pragmatic step, the outcomes from the top ten most common cancers requiring surgery were captured initially, and then two COSs from other cancer types outside the top ten (oropharyngeal and pancreatic) were added. Outcomes were limited to these 12 sets for pragmatic reasons.

For the eight cancer types (prostate^7^, colorectal^8^, gastric^3^, oesophageal^9^, oropharyngeal (ear, nose, and throat)^10^, bladder^11^, lung^12^, pancreas^13^) where COSs already existed, the core outcomes already identified were extracted from their respective publications. For the remaining cancer types for which no current COS existed (breast, liver, thyroid, and cervical), outcomes were identified and extracted from a systematic review of open and recruiting trials registered on ClinicalTrials.gov. The search was conducted on 20 March 2024, and the search strategy is described in the study protocol^18^.

### Stage 2: consolidation of outcomes

From the long lists of outcomes identified in Stage 1, a series of independent iterative reviews was undertaken by three members of the core group (JT, AL, BA), followed by discussion for each cancer type. The purpose of these reviews was to eliminate all duplicate outcomes and retain only those deemed relevant for inclusion in a meta-COS for all cancers. As an example, residual shoulder abduction deficit following breast cancer surgery would not be relevant for all cancer types, thereby would not be suitable for consideration in a surgical oncology meta-COS. Conversely, disease-free survival following cervical cancer surgery would be relevant across all cancer types, and thus would be included as a candidate outcome for the meta-COS. If there was uncertainty in the decision-making process, then the outcome was retained for consideration within the consensus stages.

### Stage 3: focus groups with key stakeholders

Three separate focus groups were organized for each stakeholder group targeting patients who have received surgery for cancer, surgeons/oncologists, and other healthcare professional who treat patients with cancer following a surgical intervention across the spectrum of different cancers (clinical nurse specialists, physiotherapists, and dieticians). The aim of the focus groups was to discuss the outcomes resulting from the consolidation stage and to provide suggestions for removing, combining, or proposing new outcomes. Alongside the patient representatives, the focus groups were also used to provide lay language on how the outcomes should be described in the consensus stages.

### Stage 4: outcome prioritization

To assist with outcome presentation and ordering, the final list of outcomes was categorized according to a 38-item taxonomy^21^ with core areas of survival, outcomes relating to cancer (adapted to ‘controlling cancer’ later in this project), physiological or clinical, life impact (following surgery), delivery of care/resource use, and adverse events. Particular attention was given to the adverse events core area because previous research suggests individual harms may be important when developing COS for targeted interventions such as surgery^22^.

Participants from each stakeholder group were asked to prioritize these outcomes in a two-round online Delphi survey. In round 1, participants were asked to score the importance of each outcome using a nine-point Likert scale with scored of 1–3 labelled not important, 4–6 labelled as important but not critical, and 7–9 labelled critically important. Participants were also given the option to select ‘I don’t know’ if they felt they could not score a particular outcome. At the end of round 1, participants were invited to submit additional outcomes, which the study group reviewed. Any suggestions representing new outcomes were added to the list to be scored in round 2. No outcomes were removed from the list between round 1 and round 2. During round 2, participants were shown the distribution of scores for each stakeholder group for each outcome, together with their own score from round 1, and asked to score the outcome again, using the same nine-point Likert scale, considering this information. The Delphi survey was managed using REDCap^23^, with round 1 open from 10 December 2024 to 20 February 2025 and round 2 open from 13 March 2025 to 1 May 2025.

### Stage 5: consensus meeting

An online consensus meeting was held virtually using Zoom (Zoom Video Communications I. (6.4.12 edn.). San Jose, CA, USA: Zoom) on 10 June 2025 and chaired by an experienced COS developer (DD). Individuals were selected to be invited to the meeting using the following broad principles: Delphi participants who completed both rounds; a balance across the three broad stakeholder groups; and a balance among those who have experience in treating or have received surgery for different cancer types. Before the meeting, participants received an indication of which outcomes had reached the predefined definition of either consensus in or consensus out in the Delphi survey (*Table 1*).

**Table 1:** Consensus criteria

Consensus classification	Description	Definition
Consensus in	Consensus that the outcome should be included in the final meta-COS	≥ 80% of participants scoring as 7–9 (critical) in each stakeholder group
Consensus out	Consensus that the outcome should not be included in the final meta-COS	≤ 50% of participants scoring 7–9 (critical) in each stakeholder group
No consensus	Uncertainty about the importance of the outcome	All other responses

COS, core outcome set.

Outcomes that reached consensus in or consensus out from the Delphi results were automatically included or excluded from the meta-COS unless there was an objection from a consensus meeting participant, in which case the outcome was discussed further. The remaining outcomes, where no consensus was reached from the Delphi process, were then prioritized for discussion, depending on how many different stakeholder groups rated them as critical for inclusion in the meta-COS. Participants were invited to briefly discuss the importance of each outcome before an anonymous vote. For the outcome to be included in the final meta-COS, 80% or more of the participants needed to vote the outcome as critical. Following the completion of the voting, the final agreed meta-COS was reviewed by the meeting participants, and a reflective discussion was undertaken to ensure the outcomes included are both pragmatic and feasible to measure for the COS’s intended purpose of use.

The 80% threshold for consensus in aligns with established COS methodology and ensures strong stakeholder agreement. Thresholds of 70–80% have been convention in Delphi consensus processes, with ≥ 70% being used for published cancer surgery COSs^3,7,10,24^. With this meta-COS aimed broadly at all surgical cancers, a higher value of 80% would ensure the COS is robust and not swayed by outlier opinions. The 50% threshold for consensus out allows for exclusion of outcomes with limited support while preventing premature elimination of potentially important outcomes.

## Results

The final meta-COS for surgical oncology is presented in *Table 2* and included eight outcomes from the adapted core areas relating to survival (four outcomes), controlling cancer (one outcome), life impact following surgery (one outcome), delivery of care (one outcome), and adverse events (one outcome).

**Table 2:** Outcomes included in the meta-core outcome set for surgical oncology

Core area	Outcome	Description
Survival	Overall survival	Length of time a patient stays alive, regardless of the cause of death, following the date of diagnosis or following the primary surgery for cancer
	Disease-free survival	Length of time a patient stays alive after the primary surgery for cancer, without any return of any signs or symptoms of that cancer
	Disease-specific survival	Length of time a patient stays alive following the date of diagnosis or following the primary surgery for cancer, when the cause of death is from the cancer itself
	Death related to surgery	Death that is caused by the cancer surgery, including the immediate postoperative period (90 days)
Controlling cancer	Completeness of tumour removal	Total removal of cancer from the body through surgery
Life impact following surgery	Overall quality of life	An assessment of how the cancer and the surgical treatment affect a patient's ability to deal with everyday life, covering physical, emotional, and social wellbeing, as well as mental health
Delivery of care	Delay to further treatment	Determination on whether any required follow-up adjuvant therapy (for example, chemotherapy or radiotherapy) or reconstruction surgery is delayed due to prolonged recovery or surgical complications following the initial primary surgery for the cancer
Adverse events	Serious adverse events	Any adverse event from the cancer surgery that has a significant impact on health or quality of life, including death

### Outcome identification

**Fig. 2:**
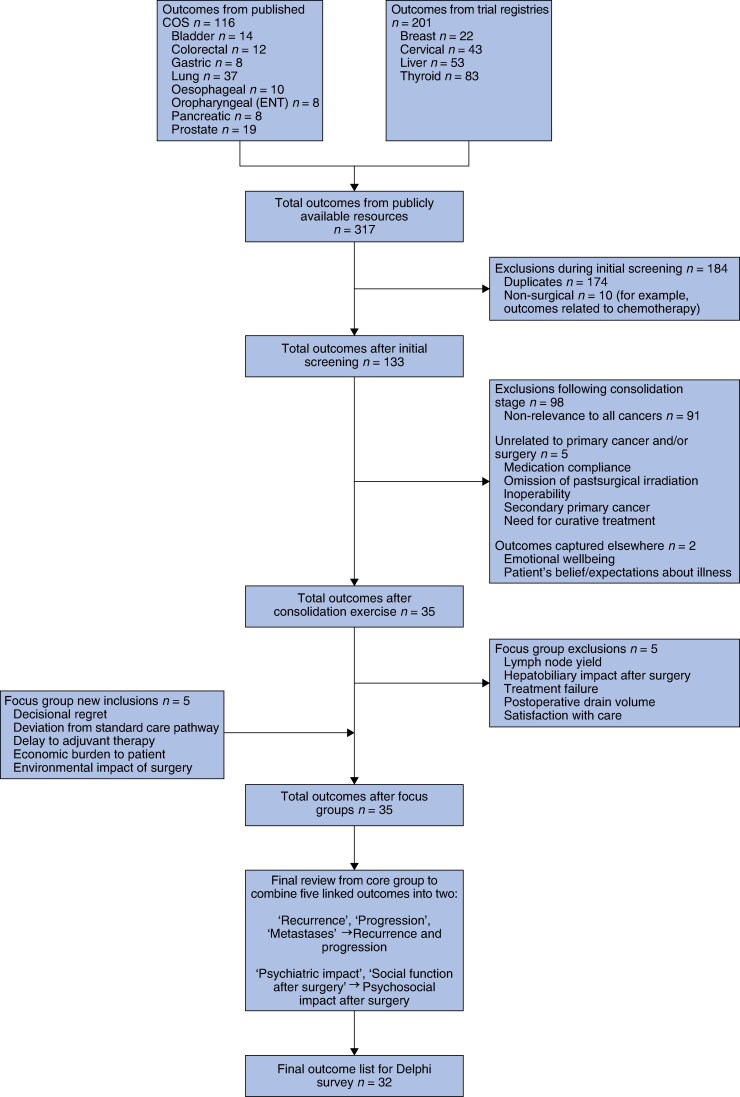
Summary of outcomes identification COS, core outcome set; ENT, ear, nose, and throat.

A summary of the outcome identification stage is provided in *Fig. 2*. In all, 317 outcomes were extracted as either core outcomes from published surgical oncology studies (116) or listed outcomes from trial registries for cancer types where COS did not exist (201). Following the initial screening and consolidation stages, the outcome list was reduced to 35 outcomes, with reductions primarily resulting from the removal of duplicate outcomes mentioned across different cancer types or outcomes deemed unsuitable for inclusion in a meta-COS of surgical oncology because they would not be relevant across all cancer types. The results from the three focus groups identified an additional five outcomes to be removed, but also suggested five new outcomes for consideration (*Fig. 2*). A final review of the remaining list of outcomes by the core group combined five outcomes into two separate outcomes with broader remit, resulting in 32 unique outcomes for use in the Delphi survey (*Fig. 2*).

### Online Delphi survey

Round 1 of the Delphi survey was completed by 47 participants, whereas 45 completed round 2 (96% retention). Participants comprised 18 patients or carers, 17 consultant oncologists/cancer surgeons and 12 specialist nurses or other allied health professionals (*Table 3*). Participants were primarily recruited from the UK and Ireland. There was at least one representative patient, consultant, and other healthcare professional from each of the 12 cancer types considered, with some patients experiencing multiple cancers and some healthcare professionals treating and caring for patients with different cancer types.

**Table 3:** Stakeholder representation for different cancer types during the Delphi survey and consensus meeting

	Patient or carer (*n*)	Consultant (*n*)	Specialist nurse or AHP (*n*)
Actual no. of stakeholders	18	17	12
**Cancer type represented**	No. in Delphi survey ( No. in consensus meeting)	No. in Delphi survey (No. in consensus meeting)	No. in Delphi survey (No. in consensus meeting)
Breast cancer	6 (2)	4 (1)	5 (1)
Lung cancer	1 (1)	1 (1)	4 (0)
Bowel cancer	3 (1)	1‡ (0)	6‡ (1)
Prostate cancer	1 (0)	3 (0)	3 (0)
Gastric cancer	2 (1)	2 (0)	6 (1)
Liver cancer	1 (1)	3 (1)	5 (1)
Cervical cancer	1 (1)	3 (1)	4 (0)
Oesophageal cancer	2 (1)	2 (0)	7 (1)
Thyroid cancer	2 (1)	2 (0)	3 (0)
Bladder cancer	1 (0)	3 (0)	4 (0)
Pancreatic cancer	2 (0)	2 (1)	5 (0)
ENT cancer	1 (0)	1 (0)	3 (0)
All cancers†			N/A (2)
**Location of participants (*n*** **=** **47)**			
Greater Manchester	6 (3)	11 (2)	8‡ (3)
Nottingham	1	0	0
Edinburgh	2 (1)	0	0
London	2	0	0
Surrey	2 (1)	0	0
Sussex	1	0	0
Newcastle	1	0	0
Liverpool	1	4‡ (2)	0
Cambridge	1 (1)	0	0
Isle of Wight	1	0	0
Republic of Ireland	0	2	4 (2)

‡Two participants did not complete round 2 of the Delphi survey. AHP, allied health professional.

At the end of round 1, two outcomes met the predefined criteria for inclusion in the meta-COS across all three stakeholder groups (*Table S1*). Three additional outcomes were suggested and added and scored by participants in round 2 following agreement by the core group, namely length of stay in hospital, social impact after surgery, and skin sensation changes after surgery. At the end of round 2, the same two outcomes had reached consensus as in round 1 (serious adverse events and overall quality of life), whereas six outcomes met the consensus criteria for exclusion (sexual function after surgery, cosmetic impact of surgery, psychological impact of surgery, environmental impact of surgery, social impact after surgery, and skin sensation changes after surgery; *Table S1*).

### Consensus meeting

Fifteen voting participants attended the consensus meeting (six patients covering five different cancers, four consultants covering four different cancers, and five allied health professionals who, combined, had experience in care for patients with all cancer types; *Table 3*). In addition, several non-voting members of the core group contributed to the discussion. No participants challenged the outcomes that were automatically included or excluded from the Delphi results. In the consensus meeting a further six outcomes met the definition for inclusion in the surgical meta-COS, in addition to the two outcomes that had reached the definition of consensus for inclusion at the end of round 2 of the Delphi survey. There were no challenges to these outcomes from the consensus meeting participants. *Table S2* provides information on the voting and final decisions from the consensus meeting alongside any salient discussion points that were made by participants when discussing outcomes.

The inclusion of the ‘delay to further treatment’ outcome under the delivery of care core area was only previously evident in the COS for lung cancer. This outcome was strongly supported in the consensus meeting (*Table S2*) and reflected the fact that any delay in the care pathway because of surgery, such as delays to adjuvant therapy from a surgical complication, may affect the likelihood of survival. The inclusion of four survival outcomes was strongly supported in the consensus meeting and reflected potential differences in life expectancy following surgery for different cancer types. The survival outcomes also address common questions that were raised about treatment decision-making from the patient participants.

## Discussion

This study used standardized methodology to develop a meta-COS for surgical oncology trials and clinical use. This overarching meta-COS represents one of the first novel attempts to span multiple cancer types in surgical oncology. It provides a list of baseline outcomes to be measured and reported for primary cancer types, such as breast, thyroid, liver, and cervical cancers, where there are no existing COSs. This is particularly beneficial because developing a COS for each cancer speciality can be time-consuming and resource intensive (the UK’s National Institute for Health Research had awarded €4.7 million to fund the development of 14 COSs^16^). Future research studies evaluating surgical oncology interventions should include the meta-COS. Although there is good harmonization between the meta-COS and existing surgical oncology COS, this study does not advocate the meta-COS developed herein as a replacement for existing surgical cancer-specific COSs. Instead, users of COSs (such as trialists) are encouraged to use the existing COS and add specific outcomes from the meta-COS not included in the original studies.

The uptake of COSs varies widely across health conditions^25^. A common barrier to uptake often relates to the lack of consensus on the instruments that should be used to assess each core outcome. Using formal assessment methods^26^ for selecting appropriate instruments to measure each core outcome was beyond the scope of this study, because specifying ‘how’ core outcomes should be measured is considered a separate, later step in the COS development process. Each cancer speciality may already have systems in place to measure outcomes, and these should be sought in the first instance, such as the European Organization for Research and Treatment of Cancer Quality of Life Questionnaire site-specific module for gastric cancer (EORTC-QLQ-STO22)^27^.

To develop a core measurement set, strict guidance is available from the COSMIN^28^ database group. Preliminary recommendations are made on the meta-COS measurement instruments. The four survival outcomes are already described and defined for cancer patients^29,30^. In the UK, information on death related to surgery and overall survival is readily collected by NHS Spine. Measures for disease-free survival and disease-specific survival will require access to cancer recurrence data through local networks.

The completeness of tumour removal can often be determined by measurement systems, such as the residual tumour (or R) classification^31^. This classification determines how complete the resection has been after surgery, with R0 denoting complete resection, R1 denoting microscopic tumour remains, and R2 denoting macroscopic tumour remains. There is a subtle variability in the description of what entails a complete resection between cancer types (for example, a 1-mm margin in rectal cancer or the absence of ink staining on tissue in breast cancer). Nevertheless, these endpoints are already collected by pathologists examining specimens from the surgery and can be complemented with pathological measures using standard data sets from The Royal College of Pathologists^32^.

The choice of quality-of-life outcome is subject to further research. The core group recommends that trialists consider validated options, with many specific tools available for each cancer surgery type, similar to the earlier example of gastric cancer. In the absence of a cancer-type-specific tool being available, the EORTC has published validated questionnaires for use in cancer trials^33^. Alternatively, the COSMIN of systematic reviews also provides available measurement tools^34^. For example, an initial search reveals a review of validated quality of life tools for oncologic breast surgery^35^.

Although the delay to further treatment outcome could be considered a process measure, it is important that it is considered at an individual cancer level. Depending on the type and stage of cancer, a patient may require further treatment modalities, such as chemoradiotherapy, chemotherapy, or immunotherapy, to cure cancer. These adjuvant options are likely to expand in the future. Any complications from the primary surgery that require postponement of the subsequent cancer treatment potentially negatively affect a patient’s survival outcome. The measurement of this delay would have to be undertaken at the site where a patient’s care is located, through the responsible cancer multidisciplinary team, which maintains an overview of cancer care.

Broadly accepted classifications for defining serious adverse events are the Clavien–Dindo classification^36,37^ or the Comprehensive Complication Index^38^. Both measure the severity and cumulative impact of all complications. An event with a Clavien–Dindo grade of III or greater would be a complication requiring an intervention, and not sufficiently treated with medications or non-invasive approaches, and one that would likely result in significant delays to the patient’s treatment pathway. This is generally accepted as a serious adverse event.

The strengths of this COS study include use of the COMET Initiative minimum recommendations for developing the meta-COS^14^, as well as guidance for protocol development^17^ and COS-STAR^19^ for reporting. The development of this meta-COS was novel. By prioritizing outcomes applicable across cancer types rather than for a specific cancer type, this trade-off between specificity and generalizability represents a pragmatic approach to addressing outcome heterogeneity in surgical oncology research.

Nevertheless, this study has a few limitations. First, although participants were recruited through national networks, the majority of participants who took part resided in the UK or Ireland, where the core study group was located. There may be different considerations in countries using primarily insurance-based healthcare systems, or in lower income countries. Despite this, the outcomes selected still reflect fundamental aspects of cancer care that transcend healthcare system boundaries.

Second, only a small number of Delphi participants attended the consensus meeting. The lack of involvement from some cancer types in this final stage was apparent and may limit the range of explored opinions. This is often a standard limitation of studies that use the Delphi methodology.

The final limitation is that although this meta-COS is planned for all cancer types, only cancers in the top ten in terms of incidence and cancers for which a current COS existed were considered as part of the COS development process, acknowledging there will be other cancer types that were not included (for example, renal and melanoma). However, these limitations are unlikely to have affected the outcome because the final results do not differ widely from the existing COSs in this field. The voting in the consensus meeting (*Table S2*) clearly indicated that these outcomes should be included or excluded from the meta-COS in accordance with the consensus criteria, with no obvious borderline decisions.

Overall, this study has developed a meta-COS in surgical oncology for use in research. This meta-COS comprises relevant outcomes that have been selected using recommended standards and are designed to be relevant and meaningful to a wide range of stakeholders, including patients who have received a surgical intervention for their cancer, regardless of cancer type. Either as stand-alone outcomes or combined with outcomes from existing cancer-specific oncology COSs, use of this meta-COS will guide and inform research by providing the opportunity to promote consistency in outcome selection. Subsequently, this will lead to the generation of more robust evidence to ensure the comparability of effectiveness across studies investigating surgical strategies for improving cancer outcomes and care. Future work must focus on seeking consensus on how the meta-core outcomes should be measured, especially for the quality-of-life measure.

## Collaborators

James Harvey (Manchester University NHS Foundation Trust, Manchester); Ashu Gandhi (Manchester University NHS Foundation Trust, Manchester); Sarah Kitson (Manchester University NHS Foundation Trust, Manchester); Hassan Malik (Aintree University Hospitals NHS Foundation Trust, Liverpool); Alex Lewis (The Christie NHS Foundation Trust, Manchester); Caroline Wilson (The Christie NHS Foundation Trust, Manchester); Peter Szatmary (Aintree University Hospitals NHS Foundation Trust, Liverpool); Rob Jones (Aintree University Hospitals NHS Foundation Trust, Liverpool); Sami Abdel Wahab (Galway University Hospital, Ireland); Vishwanath Hanchanale (Royal Liverpool University Teaching Hospital, Liverpool); Michael Shackcloth (Liverpool Heart and Chest hospital, Liverpool); Sotonye Tolofari (Northern Care Alliance, Manchester); Michael McCarthy (Galway University Hospital, Ireland); Emma Crosbie (Manchester University NHS Foundation Trust, Manchester); John Saunders (Northern Care Alliance, Manchester); Kate Williams (Manchester University NHS Foundation Trust, Manchester); Thomas Satyadas (Manchester University NHS Foundation Trust, Manchester); Amar Mohee (Manchester University NHS Foundation Trust, Manchester); Arfon Powell (Northern Care Alliance, Manchestern); Matthew Evison (Manchester University NHS Foundation Trust, Manchester); Michelle Harvie (Manchester University NHS Foundation Trust, Manchester); Neil Bibby (Manchester University NHS Foundation Trust, Manchester); Ashleigh Maske (The Christie NHS Foundation Trust, Manchester); Kellie Owen (Northern Care Alliance, Manchester); Sophie Peirson (Manchester University NHS Foundation Trust, Manchester); Fionnuala Ginty (Galway University Hospital, Ireland); Meghan Carter (Galway University Hospital, Ireland); Kathryn Ellis (Manchester University NHS Foundation Trust, Manchester); Helen Molloy (Northern Care Alliance, Manchester); Sarah Gallagher (The Christie NHS Foundation Trust, Manchester); Maria Kikayi (The Christie NHS Foundation Trust, Manchester); Catherine Masterson (Galway University Hospital, Ireland); Grainne McDonnell (Galway University Hospital, Ireland); Lauren Woods (Northern Care Alliance, Manchester) Hilary Stobart; Helen Bulbeck; John Crouchley; Margaret Grayson; Elizabeth McCall; Ceri Steele; Curie Freeborn; Lisa Crane; Adrienne Morgan; Jo Saunders-Betts; Dave Chuter; Alison Tait; Amy Heley; Gim Bee Yeoh; Ciaran McIntyre; Susanne Barrett; Nicholas Clews; Janice Brown; Simona Smutna; Geoff Libby.

## Supplementary Material

zrag019_Supplementary_Data

## Data Availability

Data relating to the search strategy are published in the protocol paper (https://hrbopenresearch.org/articles/8-19), which is open access. The results of the Delphi and consensus meeting scores are summarized in the Supplementary material in this open access publication. Transcripts of the consensus meeting and voting results are stored in The University of Manchester secure OneDrive and are accessible only to members of the project management team.
