# Supplementary material for: Meta-core outcome set for randomized trials and clinical practice for curative surgical oncology: patient and healthcare consensus statement
Source: BJS Open. 2026 Mar 24;10(2):zrag019. doi: 10.1093/bjsopen/zrag019 (PMC13010334; doi:10.1093/bjsopen/zrag019)
Supplement: zrag019_Supplementary_Data [file zrag019_supplementary_data.docx]

***A meta-core outcome set for randomised trials and clinical practice for curative surgical oncology: a patient and healthcare consensus statement***

Joel Tay^1^, Jane Blazeby^2^, Declan Devane^3,4^, Yoon Loke^5^, Aoife Lowery^6^, Adam O’Neill^7^, Catherine Robinson^7^, Ceri Steele^8^, *Bilal Alkhaffaf^9,10^ & *Jamie Kirkham^1^

*Joint senior authors

^1^Centre for Biostatistics, The University of Manchester, Manchester Academic Health Science Centre, Manchester, United Kingdom.

^2^NIHR Bristol Biomedical Research Centre, University Hospitals Bristol and Weston NHS Foundation Trust and University of Bristol

^3^Health Research Board-Trials Methodology Research Network (HRB-TMRN)

^4^School of nursing and Midwifery, University of Galway, Galway, Ireland University of Galway, Galway, Ireland

^5^Norwich Medical School, University of East Anglia, UK

^6^Discipline of Surgery, University of Galway, Ireland

^7^Social Care and Society, School of Health Sciences, The University of Manchester, United Kingdom

^8^Patient representative of Independent Cancer Patients’ Voice (ICPV)

^9^ Faculty of Biology, Medicine and Health, The University of Manchester, Manchester Academic Health Science Centre, Manchester, United Kingdom.

^10^Department of Oesophago-Gastric and Bariatric Surgery, Salford Royal Hospital, Northern Care Alliance NHS Foundation Trust, Salford, UK. Division of Cancer Sciences, School of Medical Sciences, Faculty of Biology, Medicine and Health, University of Manchester, Manchester, UK

**^*^Corresponding Author:**

Joel Tay

Centre for Biostatistics

Faculty of Biology, Medicine and Health

The University of Manchester

Jean McFarlane Building

Oxford Road

Manchester, M13 9PL, UK

Email: joel.tay@doctors.org.uk

[**https://orcid.org/0000-0002-6939-3442**](https://orcid.org/0000-0002-6939-3442)

X: @JoelWTTay

**Supplementary Materials - Index**

| **Supplementary Appendixes** |  |
| --- | --- |
| COS-STAR checklist | *Manuscript page 4; Methods* |
| **Supplementary Figures and Tables** |  |
| Table 1 | *Manuscript page 10; Results* |
| Table 2 | *Manuscript page 10; Results* |

**Supplementary Appendix**

**Core Outcome Set-STandards for Reporting: The COS-STAR Statement Checklist**

| **SECTION/TOPIC** | **ITEM No.** | **CHECKLIST ITEM** | **REPORTED ON PAGE NUMBER** |
| --- | --- | --- | --- |
| TITLE/ABSTRACT | | | |
| Title | 1a | Identify in the title that the paper reports the development of a COS | 1 |
| Abstract | 1b | Provide a structured summary | 2 |
| INTRODUCTION | | | |
| Background and Objectives | 2a | Describe the background and explain the rationale for developing the COS. | 3 |
|  | 2b | Describe the specific objectives with reference to developing a COS. | 3 |
| Scope | 3a | Describe the health condition(s) and population(s) covered by the COS. | 3 |
|  | 3b | Describe the intervention(s) covered by the COS. | 3 |
|  | 3c | Describe the setting(s) in which the COS is to be applied. | 3-4 |
| METHODS | | | |
| Protocol/Registry Entry | 4 | Indicate where the COS development protocol can be accessed, if available, and/or the study registration details. | 5 |
| Participants | 5 | Describe the rationale for stakeholder groups involved in the COS development process, eligibility criteria for participants from each group, and a description of how the individuals involved were identified. | 5-6 |
| Information Sources | 6a | Describe the information sources used to identify an initial list of outcomes. | 6-7 |
|  | 6b | Describe how outcomes were dropped/combined, with reasons (if applicable). | 7-8 |
| Consensus Process | 7 | Describe how the consensus process was undertaken. | 8-9 |
| Outcome Scoring | 8 | Describe how outcomes were scored and how scores were summarised. | 8-9 |
| Consensus Definition | 9a | Describe the consensus definition. | 8-9 |
|  | 9b | Describe the procedure for determining how outcomes were included or excluded from consideration during the consensus process. | 8-9 |
| Ethics and Consent | 10 | Provide a statement regarding the ethics and consent issues for the study. | 5 |
| RESULTS | | | |
| Protocol Deviations | 11 | Describe any changes from the protocol (if applicable), with reasons, and describe what impact these changes have on the results. | N/A |
| Participants | 12 | Present data on the number and relevant characteristics of the people involved at all stages of COS development. | 17,18,21 |
| Outcomes | 13a | List all outcomes considered at the start of the consensus process. | 25 |
|  | 13b | Describe any new outcomes introduced and any outcomes dropped, with reasons, during the consensus process. | 25 |
| COS | 14 | List the outcomes in the final COS. | 20 |
| DISCUSSION | | | |
| Limitations | 15 | Discuss any limitations in the COS development process. | 12-13 |
| Conclusions | 16 | Provide an interpretation of the final COS in the context of other evidence, and implications for future research. | 14-15, 22 |
| OTHER INFORMATION | | | |
| Funding | 17 | Describe sources of funding/role of funders. | 18 |
| Conflicts of Interest | 18 | Describe any conflicts of interest within the study team and how these were managed. | 18 |

*From: Kirkham JJ, Gorst S, Altman DG, Blazeby JM, Clarke M, Devane D, et al. (2016) Core Outcome Set–STAndards for Reporting: The COS-STAR Statement. PLoS Med 13(10): e1002148. https://doi.org/10.1371/journal.pmed.1002148*

**Supplementary Table 1: Comparison of Delphi survey Round 1 and Round 2 results by stakeholders**

|  | Percentage scored 7-9 | | Percentage scored 7-9 | | Percentage 7-9 | |
| --- | --- | --- | --- | --- | --- | --- |
|  | R1 consultants | R2 consultants | R1 Nurses and AHPs | R2 Nurses & AHPs | R1 Patients & carers | R2 Patients & carers |
| **Survival outcomes** |  |  |  |  |  |  |
| Disease-specific survival | 82% | 81% | 75% | 73% | 78% | 72% |
| Disease-free survival | 82% | 81% | 75% | 55% | 83% | 83% |
| Overall survival | 88% | 94% | 50% | 45% | 78% | 78% |
| Death related to surgery | 88% | 94% | 75% | 73% | 67% | 67% |
| **Controlling cancer outcomes** |  |  |  |  |  |  |
| Progression or recurrence | 76% | 75% | 67% | 82% | 94% | 89% |
| Completeness of tumour removal | 82% | 88% | 83% | 73% | 94% | 89% |
| **Delivery of care outcomes** |  |  |  |  |  |  |
| Resource use | 47% | 44% | 58% | 55% | 72% | 67% |
| Delay to further treatment | 65% | 63% | 83% | 64% | 78% | 72% |
| Deviation from care pathway | 53% | 31% | 75% | 45% | 78% | 61% |
| **Adverse event outcomes** |  |  |  |  |  |  |
| Anastomotic leak | 65% | 56% | 58% | 45% | 67% | 56% |
| Blood loss | 47% | 44% | 42% | 18% | 72% | 56% |
| Rate of stoma formation and related impacts | 59% | 56% | 50% | 36% | 72% | 67% |
| Surgical site infections | 35% | 38% | 42% | 27% | 50% | 67% |
| Adverse events | 65% | 75% | 58% | 45% | 78% | 78% |
| Serious adverse events | 82% | 94% | 83% | 91% | 94% | 89% |
| Venous thromboembolism | 35% | 25% | 50% | 45% | 78% | 72% |
| **Impacts of surgery outcomes** |  |  |  |  |  |  |
| Overall quality of life | 88% | 94% | 83% | 82% | 83% | 89% |
| Ability to return to ADLs or work | 94% | 94% | 75% | 64% | 72% | 78% |
| Gastrointestinal impact of surgery | 41% | 44% | 58% | 55% | 61% | 50% |
| Respiratory impact of surgery | 41% | 38% | 58% | 45% | 78% | 72% |
| Nutritional impact of surgery | 41% | 44% | 75% | 73% | 67% | 61% |
| Physical impairment after surgery | 65% | 69% | 75% | 73% | 83% | 78% |
| Sexual function after surgery | 53% | 44% | 50% | 27% | 44% | 39% |
| Urinary function after surgery | 53% | 44% | 42% | 18% | 67% | 61% |
| Cognitive function after surgery | 71% | 50% | 58% | 45% | 78% | 72% |
| Cosmetic impact of surgery | 41% | 25% | 42% | 18% | 33% | 28% |
| Pain | 59% | 50% | 50% | 45% | 72% | 67% |
| Sleep disturbance | 41% | 31% | 42% | 36% | 56% | 56% |
| Psychological impact after surgery | 65% | 38% | 58% | 27% | 72% | 28% |
| Environmental impact of surgery | 29% | 25% | 8% | 0% | 17% | 6% |
| **Other outcomes** |  |  |  |  |  |  |
| Economic burden to patient and carer | 53% | 50% | 58% | 55% | 56% | 50% |
| Decisional regret | 82% | 75% | 75% | 55% | 44% | 44% |
| Length of stay in hospital | N/A | 19% | N/A | 55% | N/A | 33% |
| Social impact after surgery | N/A | 38% | N/A | 36% | N/A | 39% |
| Skin sensation changes after surgery | N/A | 6% | N/A | 9% | N/A | 28% |

**Supplementary Table 2:** **Voting decisions and key discussion points during consensus meeting**

***80% and above*** scoring ***critically important*** indicates the outcome should be in the final core outcome set.

|  | **Surgical Oncology Outcomes** | **No. scored “Not important”** | **No. scored “Important”** | **No. scored “Critical”** | **% critical** | **Comment(s) extracted from transcript** | **Decision** |
| --- | --- | --- | --- | --- | --- | --- | --- |
| **Survival theme/outcome domain** | | | | | | | |
| 1 | Disease-free survival | 0 | 2 | 13 | 87% | - | Include |
| 2 | Disease-specific survival | 0 | 2 | 12 | 86% | **Patient** (lung): surprised that this was not included automatically from the Delphi.  **Consultant** (HPB): This is one of the most common questions we get from patients.  *Single voter absent, but does not affect result*. | Include |
| 3 | Death-related to surgery | 0 | 3 | 12 | 80% | *Required third vote as individual absent and borderline result*  **Consultant** (lung): Important to capture even if death could be related to cancer process itself, like recurrence, as if a patient has had surgery in the last 90 days, it reflects a problem with the patient selection process. | Include |
| 4 | Overall survival | 1 | 1 | 13 | 87% | **Consultant** (lung): Important to include as captures impact of other co-morbidities | Include |
| **Controlling cancer theme/outcome domain** | | | | | | | |
| 5 | Progression or Recurrence | 7 | 4 | 4 | 27% | **Consultant**(breast): This is agreed to be important, but would be captured in ‘disease-free survival’ outcome.  **Consultant** (HPB): The measurement of this would be difficult | Exclude |
| 6 | Completeness of tumour removal | 0 | 2 | 13 | 87% | **Patient** (lung): The impact of radiotherapy was much greater than surgery, where the margins were not clear. So this is right up there (important). | Include |
| **Delivery of care theme/outcome domain** | | | | | | | |
| 7 | Deviation from care pathway | 11 | 3 | 1 | 7% | **Consultant** (breast): This is dependent on many factors, like which care pathway, which country, and team-dependent.  **Consultant** (lung): Uptake of adjuvant therapy would be a better outcome to measure. | Exclude |
| 8 | Resource use | 5 | 6 | 4 | 27% | - | Exclude |
| 9 | Delay to further treatment | 0 | 1 | 14 | 93% | - | Include |
| **Adverse event theme/outcome domain** | | | | | | | |
| 10 | Blood loss | 12 | 2 | 1 | 7% | **Consultant** (HPB): This is not important for all surgery, and the volumes are different for different cancer surgeries.  **Consultant** (Lung): The need for blood transfusion would be more important, as losing 50 and 150mls of blood has little effect on surgical outcome. | Exclude |
| 11 | Surgical site infections | 7 | 71 | 1 | 7% | **Consultant** (breast): If this infection affected further treatments it might be more relevant.  Patient (gastric): This is important to me even if it is not reflected in the voting into a COS. | Exclude |
| 12 | Venous thromboembolism | 12 | 2 | 1 | 7% | **Physiotherapist** (all cancers): This is not an outcome related to the surgery itself and could be more to do with patient co-morbidity.  **Consultant** (breast): This have very little impact from a cancer (outcome) perspective. | Exclude |
| 13 | Anastomotic leak | 12 | 2 | 1 | 7% | **Physiotherapist** (all cancers): Not relevant to non-gastrointestinal cancer types | Exclude |
| 14 | Rate of stoma formation and related impacts | 12 | 2 | 1 | 7% | - | Exclude |
| 15 | Adverse events (any) | 6 | 7 | 2 | 13% | **Consultant** (UGI): This would be captured in overall quality of life, and *serious* adverse event is already included in the final COS | Exclude |
| **Impact of surgery theme/outcome domain** | | | | | | | |
| 16 | Gastrointestinal impact of surgery | 10 | 4 | 1 | 7% | **Consultant** (breast): Not relevant to non-gastrointestinal cancer types  **Consultant** (HPB) The more serious symptoms would be captured in ‘serious adverse events’ outcome, e.g. Clavien-Dindo grade 3+. | Exclude |
| 17 | Respiratory impact of surgery | 10 | 5 | 0 | 0% | **Consultant** (HPB): Important to note that patients felt strongly that this is important but at the same time recognised it was not applicable to all surgical cancer-types | Exclude |
| 18 | Urinary function after surgery | 13 | 2 | 0 | 0% | - | Exclude |
| 19 | Cognitive function after surgery | 4 | 8 | 3 | 20% | **Consultant** (breast): this is more commonly seen in the some of the other therapies for cancer, rather than the actual surgery  Patient (lung): Some experience cognitive dysfunction after a general anaesthetic and not quite the same  Patient (thyroid & breast): My mother had brain damage from surgery many years ago thought this should have been included. | Exclude |
| 20 | Pain | 5 | 6 | 4 | 27% | **Patient** (liver): For a patient to report this it probably is a significant pain as they are inclined o build a pain tolerance already. There might be a disconnect between consultants, nurse specialists and patients.  **Consultant** (HPB): This pain would also be captured under ‘Overall quality of life’ outcome for which there is already a score system.  **Consultant** (breast): Need for opiate use might be more suitable, as evidenced by some anaesthetic journals. **Patient** (breast): This would be hard to measure as pain thresholds can be subjective to individuals.  **Patient** (liver): It’s important not to measure just what would be easy to measure as it will be a ‘beige’ outcome and missed the criticality. | Exclude |
| 21 | Sleep disturbance | 11 | 4 | 0 | 0% | **Patient** (gastric): This is important but could be a combination of anxiety, hospitalisation, ‘institutionalisation’ and not just caused by the surgery, it certainly goes beyond the surgery. | Exclude |
| 22 | Economic burden to patient and carer | 1 | 11 | 3 | 20% | **Consultant** (HPB): Global consideration is needed as this outcome might be more relevant outside of NHS,UK for insurance-based healthcare systems, if we are to present this as a COS for all to use.  Patient (lung): some patients are eligible to claim benefits and welfare, and so there is still health inequalities related to this in the UK.  **Consultant** (breast): a patient lost their home after cancer treatment in the UK, due to the impact on a home’s main breadwinner. But I am unsure whether we should be reporting this for every single cancer.  **Consultant** (UGI): This would be important to write about in the thesis as it was voted 50-55% in Delphi for each stakeholder group.  **Patient** (liver): More weightage (in votes) should be given to those who cannot afford healthcare, or have had a financial impact from cancer treatment, than those who can afford or in the NHS, UK. | Exclude |
| 23 | Length of stay in hospital | 7 | 4 | 4 | 27% | - | Exclude |
| 24 | Nutritional impact of surgery | 9 | 3 | 3 | 20% | **Consultant** (breast): This is not relevant to non-gastrointestinal cancers, and can relate to cancer itself. | Exclude |
| 25 | Physical impairment after surgery | 1 | 9 | 5 | 33% | - | Exclude |
| 26 | Ability to return to activities of daily living (ADL) or work | 0 | 7 | 8 | 53% | - | Exclude |
| **Other** | | | | | | | |
| 27 | Decisional regret | 2 | 10 | 3 | 20% | **Patient** (liver): We have no means of knowing what patients’ (who have not survived) regret might have been. Consultants’ might have a broader picture of regret.  **Consultant**: this is important topic, however it is difficult to ascertain if the regret is to do with surgery or the other therapies  **Consultant** (breast): I see this a lot in my practice. This can come down to when the patient is asked in the treatment process. My answer at 1 month from surgery would be different to 3 years after.  **Patient** (breast): I get asked to comment on decisional regret forms. Just by asking whether you are going to regret, is going to change the way patients think. Also regret when given a choice of surgery or radiotherapy is a different regret, that is also important. | Exclude |
